# Dynamic backgrounds enhance the confusion effect

**DOI:** 10.1098/rsos.261915

**Published:** 2026-10-28

**Authors:** Luca C.R. Courtney, Innes C. Cuthill, Ute Leonards, Nicholas E. Scott-Samuel

**Affiliations:** ^1^ School of Psychology and Neuroscience, University of Bristol, Bristol, UK; ^2^ School of Biological Sciences, University of Bristol, Bristol, UK

**Keywords:** motion camouflage, predator–prey interactions, prey aggregation, defensive colouration, group living

## Abstract

Natural environments are generally dynamic; examples of background motion include changes in illumination and moving physical features, such as wind-blown foliage or flowing water. Experimental evidence suggests dynamic backgrounds can impede predation by masking the movement of a target, but the interaction between background motion and the movement of prey in groups has not been investigated. Dynamic backgrounds could potentially enhance the confusion effect, the phenomenon whereby locating and/or tracking a single target among similar individuals (‘distractors’) becomes less successful owing to difficulty in discriminating this target from its conspecifics. We investigated whether dynamic backgrounds contribute to the confusion effect by impeding accurate tracking of a target among distractors. We hypothesize that any effect would be greatest when the temporal properties of the background's movement match those of the moving objects. In two experiments, human surrogate ‘predators’ tracked a single target among distractors, moving unpredictably, on backgrounds that drifted coherently in a given direction. Tracking was generally poorer when backgrounds and objects moved at more closely matched speeds, probably owing to temporary masking of prey target movement and subsequent difficulty in re-locating the target among distractors. Our findings identify a role for dynamic backgrounds in defending against predation.

## 1. Introduction

Motion presents a significant risk to prey, capturing predator attention and facilitating detection [1–3], as well as breaking background matching camouflage [4,5]. However, motion may also reduce predation risk beyond allowing prey to avoid predators and flee when under pursuit. This paper considers two factors relevant to this risk for group-living organisms: the movement of conspecifics and motion in the background.

Although larger groups of animals are more conspicuous and readily detected [6,7], group movement can provide a significant advantage under attack. One of these is the confusion effect: reduced attack success in predators resulting from difficulty in discriminating a target prey animal from similar non-target conspecifics (‘distractors’) [8]. Experimental evidence with human ‘predators’ demonstrates a reduction in tracking accuracy as the numerosity and density of groups increase [9,10]. In fish, increased group size and density result in increased targeting error in predators [11,12] and prey fish preferentially join larger and denser shoals [13]. The confusion effect is thought to be widespread, particularly for visual predators with agile prey [14], and is attributed to cognitive bottlenecks and constraints at numerous levels of perceptual processing [15,16].

In addition to the possibility of confusion between similar moving prey, once an individual animal is identified as a target, the other individuals become a background from which this target must be discriminated [17]. Hall *et al.* [18] found that, while individual moving targets were easily identified irrespective of patterning, identification of a camouflaged target was slowed by the presence of moving distractors with similar patterning, and this benefit in reduced detection was greater as the number of distractors increased. The presence of moving distractors can mitigate the cost of motion to a target by increasing the amount of surrounding visual noise (i.e. reducing the signal-to-noise ratio [19]), thereby impeding predation.

Motion noise can also arise from dynamic elements in the environments themselves. Indeed, motion is pervasive in natural environments [3], arising from physical features, such as wind-blown foliage in the background [20–24], or the medium in which animals move, such as flowing rivers or tidal movement in the sea [3]. Dynamic illumination is another common phenomenon, where light passing through physical features results in varying illumination patterns on a substrate. Light filtering through moving foliage results in dappled light patterns on terrestrial surfaces [25]. In aquatic environments, the diffraction of light as it passes through the water surface results in caustic patterns [26–28]. These patterns of dynamic illumination vary spatially and temporally and are therefore a significant source of motion noise in a range of natural environments. Matchette *et al.* [25] found that human participants were poorer at capturing a moving target in scenes with either simulated water caustics or dappled forest light, suggesting that dynamic illumination can mask a target's movements and impede detection. This result was replicated in subsequent experiments with domestic fowl (*Gallus gallus domesticus*; [29]), Picasso triggerfish (*Rhinecanthus aculeatus*; [30]) and three-spined sticklebacks (*Gasterosteus aculeatus*; [31]). Dynamic backgrounds may therefore be an impediment to successful predation across taxa. Evidence that fish predators avoid environments with dynamic illumination [31] gives weight to this proposition.

Indeed, animals appear attuned to the dynamic properties of their environment and adapt their behaviour in response. Vine snakes (*Oxybelis aeneus*) and stick insects (*Phasmatidae*) display swaying or oscillating movements when presented with visual cues of wind, with a frequency similar to that of surrounding foliage [32–34], which may help the animal conceal itself against the motion of its background. For movement-based signals, the disruptive effects of motion noise in the background on detectability are greatest when this matches the temporal properties of the signal (i.e. speed and frequency) [21,22,24]. Logically, this principle should apply to the corresponding effect in predation scenarios, where matching the temporal properties of a coherent dynamic background may minimize the visual signal-to-noise ratio [19,22], impeding tracking more effectively. While such a strategy has been observed for individual animals, we propose that this principle should be particularly important for animals moving in groups, to reduce the greater salience to predators that aggregation brings.

Here, we tested whether dynamic visual scenes interact with the confusion effect, hindering successful tracking of individual, background matching, moving targets among similar distractors. We adapted the computer-based tracking task of Scott-Samuel *et al.* [10] with humans acting as surrogate predators. In two experiments, participants had to track a single target object among distractors: first, we investigated the influence across group sizes of dynamic backgrounds moving at different speeds, to establish whether dynamic backgrounds enhance the typical increase in confusion with group size; and second, we assessed the importance of a match between object and background speed, to clarify the nature of any masking effect. The visual object tracking task was presented on drifting backgrounds—a coherent, directed background pattern relating to a different class from that considered in many previous studies, that is, the undirectional quasi-chaotic motion noise caused by water caustics [25,30,31] or the oscillating movement of vegetation in a breeze [21,32,34]. This is a task which plausibly arises in predator–prey encounters across natural environments. Examples include: avian predators, such as plunge-divers like kingfishers (Alcedinidae, Cerylinae) and kiskadees (*Pitangus* spp.) or wading birds like herons (Ardeidae), tracking shoaling fish prey in flowing rivers and tidal waters [35–38]; freshwater piscine predators, such as *Crenicichla* and *Micropterus*, foraging in a current made turbid by particulate matter [39,40], also see [41]; flying targets moving against a background of falling snow or rain; and insectivorous predators in arid and littered terrestrial environments with directional air currents [20], for instance, depredation of ants by desert horned lizards (*Phrynosoma platyrhinos*) and some other iguanids [42,43] and of locusts (*Schistocerca gregaria*) by their diverse avian and terrestrial predators [44].

As such, our study aims to broaden understanding of how motion noise affects predation success in real environments. We hypothesize that tracking accuracy would be significantly reduced in trials with moving rather than static backgrounds and additionally that tracking would be least accurate when the speed of objects is the same as the speed of background drift.

## 2. Method

### 2.1. Experiment 1

#### 2.1.1. Participants

Twenty students at the University of Bristol (15 female and five male, age: *M* = 20.8, s.d. = 1.01 years) were recruited opportunistically as naïve participants. This sample size gives us comparable statistical power to detect effects such as those observed by [9,10], with expected similar variance within and between participants owing to similar designs and participant pool. Each participant gave their informed written consent in line with the Declaration of Helsinki, and the research was approved by the School of Psychological Science Student Research Ethics Committee (ref: 25979). Participants were reimbursed with Amazon vouchers worth £7.

#### 2.1.2. Stimuli

The experiment was created in MATLAB R2024b Version 24.2.0.2740171 Update 1, using the Psychophysics Toolbox Version 3.0.19 extensions [45,46]. Stimuli were presented on a 15.6“ Huawei Matebook D15 laptop screen, with a temporal resolution of 60 Hz and a spatial resolution of 1920 × 1080 pixels. Viewing distance was 60 cm and therefore each pixel subtended 1.03 arc minutes.

The task required participants to visually track a single moving target among similar moving distractors for a period of 6 s and subsequently identify its final location on the screen via mouse click. Sets of either 20, 40 or 80 squares (containing one target, the rest distractors) were placed randomly within a central area of the screen, measuring 512 × 512 pixels (13.55 × 13.55 cm^2^). Squares measured 32 × 32 pixels (0.55 visual degrees) and moved at a speed of 4 pixels per frame (240 pixels s^–1^ and 4.12 deg s^–1^). The direction of each square's movement was independent of the others and was a correlated random walk, determined by values randomly drawn from a circular Gaussian distribution on each frame centred on the previous frame's direction, such that continuing in the same direction was most likely and large deviations less likely. The s.d. of this distribution was set at π/2^3.5^ rad. The speed and unpredictability of object movement were informed by values used in [10] and a pilot experiment. The leading edge of each square remained the same (i.e. squares rotated as their heading changed). Upon reaching the border, the targets would bounce off it elastically, with the angle of reflection equalling the angle of incidence. The target object was plotted in front of distractor objects, meaning it was never occluded by any other object.

Figure 1 illustrates the background and object (target and distractor) stimuli. Both the background and objects were constructed from square noise elements measuring 4 × 4 pixels (0.07 deg), each of which was randomly dark grey (value = 63 on the display greyscale range of 0 to 255) or light grey (value = 191) with probability 0.5. The target and distractors had a perfectly background matching binary noise texture. Drifting motion was generated by creating a background texture far larger than the square aperture and drifting the aperture in the opposite direction from the desired direction of movement (e.g. moving the aperture downwards creates a background that drifts upwards). The aperture itself always remained central on the screen.

**Figure 1.**
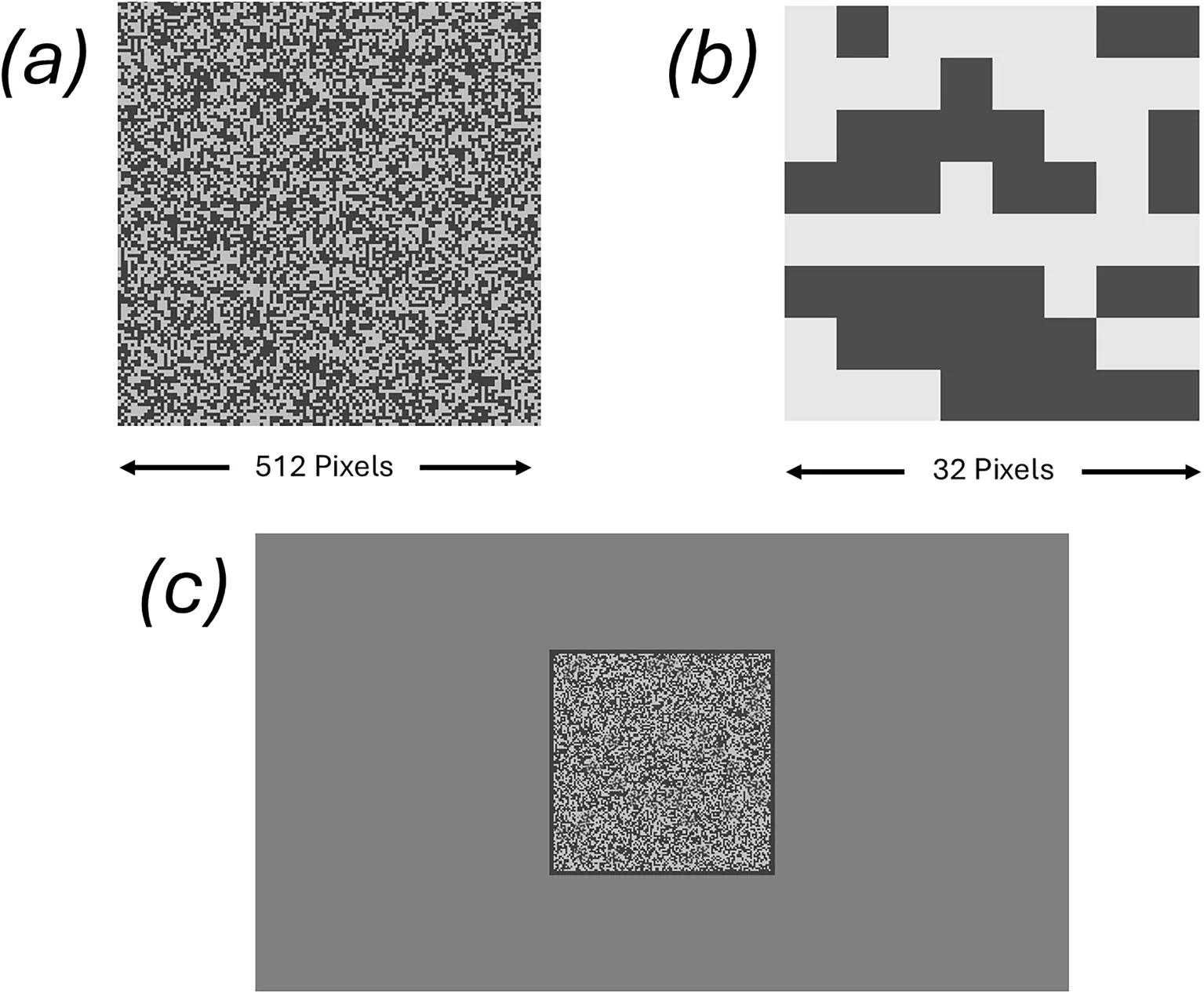
Illustration of experimental stimuli. (*a*) Background, consisting of a binary noise texture. Individual noise elements (dark and light) measured 4 × 4 pixels. (*b*) Target object, with background matching binary noise texture. (*c*) Stimulus displayed on-screen, with the binary noise background in the central area. The target and distractors moved on this background and were barely visible when static (as here) because they were background matching.

Four manipulations were applied to the speed at which the background moved: it drifted in a given direction at either 2 pixels per frame (120 pixels s^–1^ and 2.06 deg s^–1^), 4 pixels per frame (240 pixels s^–1^ and 4.12 deg s^–1^) or 8 pixels per frame (480 pixels s^–1^ and 8.24 deg s^–1^), or was static. Drift directions were leftwards, rightwards, upwards or downwards in any one trial, randomly varying from trial to trial but in equal proportions across a participant's complete experiment. Therefore, in the drifting conditions, the background moved at either half the speed, identical speed or double the speed at which the objects moved. Note, importantly, that objects moved in random directions while the background motion was always unidirectional within a trial.

The experimental design was a 3 × 4 factorial repeated-measures design, with object group size (20, 40 and 80 objects) and background drift speed (0, 2, 4 and 8 pixels per frame) as independent variables. Tracking success was operationalized as correct identification of the target (‘hit’) at the end of a trial.

#### 2.1.3. Procedure

Participants were instructed to track a target object, identified by a red annulus at the beginning of a trial, and indicate its final location with a mouse click (figure 2). At trial onset, objects moved randomly within the central area on-screen for 6 s. The target was bordered by a red annulus for the first 1 s, which then disappeared for the remaining 5 s. Next, the background became grey, and each object was replaced by a black, bounding annulus. Participants were instructed to select the circle at the final location of the designated target object using a red trackpad-driven dot. The Euclidean distance, in pixels, between the centre of the target object and the centre of the cursor upon clicking was recorded on each trial.

**Figure 2.**
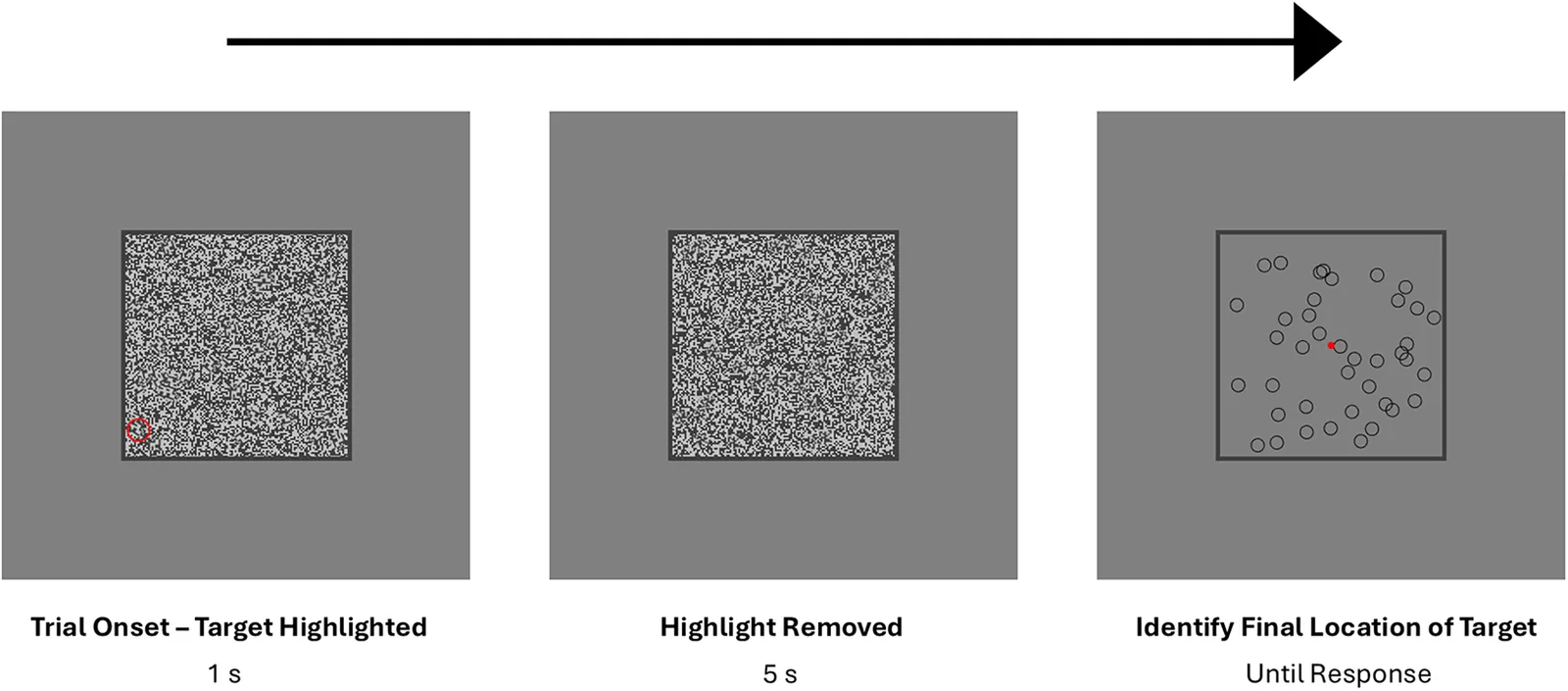
Illustration of a single trial. Objects moved randomly within the central area for 6 s. After the moving period, objects disappeared and were replaced by black circles, and participants identified the target object. Backgrounds were either static or drifted in one of the four cardinal directions, at speeds of 2, 4 or 8 pixels per frame (supplementary material, videos 1 and 2).

Each participant completed 192 trials in total, in four blocks. Each of the 48-trial blocks contained each of the three group sizes, in each of the four directions of background drift, at each of the four levels of drift speed, randomly ordered. Opportunities for short breaks were presented both halfway through each block and between blocks. The experiment took approximately 35 minutes to complete.

#### 2.1.4. Statistical analysis

Statistical analyses were conducted using R 4.5.2 [47] implemented in RStudio 25.09.0 [48], with graphs generated using the ‘ggplot2’ package [49] and the colourblind-accessible Okabe–Ito palette [50]. The threshold for coding error values as ‘hit’ or ‘miss’ was determined as 16 pixels (0.27 visual degrees) in Euclidean distance from the centre of the target object, which was the radius of the circles that the target and distractors were replaced by after the moving period. Overall, 48.5% of targets were hits and 51.5% were misses. Data were then checked for any participants with an unusually high number of misses, indicating a failure to engage with or an inability to perform the task; there were none, and the between-subject variation was approximately normally distributed (Shapiro–Wilk: *w* = 0.95, *p* = 0.415).

Generalized linear mixed-effects models (GLMM) with a binomial error and logit link function, using the glmer function in the ‘lme4’ package [51], were fitted to the hit/miss data to establish the main effects and interaction of group size and background drift speed, each modelled as factors. An analysis of deviance table with type II sums of squares was obtained using the anova function in the ‘car’ package [52], with *p*-values obtained by comparison to chi-squared distributions. In all models, a maximal random-effects structure was used (see [53]) for participant with random intercepts and random slopes for group size and drift speed, and their interaction. From previous studies of the confusion effect (e.g. [10]), the proportion of errors was expected to increase with each doubling of object number, so group size was modelled with polynomial contrasts to allow assessment of the linearity or otherwise of the relationship. Our hypothesis about the effect of background drift speed was not based on prior empirical tests (there are none), so post hoc pairwise comparisons were conducted using Tukey-style tests, with the glht function in the ‘multcomp’ package [54]. Note that, for these post hoc tests, an intercepts-only random-effects structure was fitted, as models including random intercepts and slopes were singular with very small variance estimates for the random slopes.

### 2.2. Experiment 2

Twenty-four naïve participants (19 female and five male, Age: *M* = 19.88, s.d. = 1.57 years old) were recruited through self-sign-up via the School of Psychological Science's Experimental Hours scheme and were reimbursed in course credit (Ethics Committee ref: 27781). Each gave their informed written consent in line with the Declaration of Helsinki. The display set-up and equipment were identical to those used in Experiment 1, and the tracking task was adapted for the aims of the current experiment: to establish whether effects in Experiment 1 were related to a particular background drift speed or to a match between the latter and object speed.

Forty objects (one target, 39 distractors) were presented within the central area on-screen in all trials, with all stimulus attributes identical to those used in the previous experiment, except for the range of background and object speeds. Objects moved at one of three speeds in any given trial: 2 (120 pixels s^–1^ and 2.06 deg s^–1^), 4 (240 pixels s^–1^ and 4.12 deg s^–1^) or 6 pixels per frame (360 pixels s^–1^ and 6.18 deg s^–1^), varying between trials only. Backgrounds drifted at one of three speeds in any given trial: 2, 4 or 6 pixels per frame. The departure from the octave-stepped (2, 4 and 8 pixels per frame) speed values of Experiment 1 was necessary as pilot experiments suggested that tracking was virtually impossible when objects moved at 8 pixels per frame, which would have obscured any potential treatment effects.

Experiment 2 adopted a 3 × 3 factorial repeated measures design, with object speed (2, 4 and 6 pixels per frame) and background drift speed (2, 4 and 6 pixels per frame) as independent variables. As in Experiment 1, the dependent variable was error, operationalized as hits or misses.

The procedure was similar to that of the previous experiment. Each participant completed 180 trials in total, in three blocks. Within each block, five trials were completed for each of the three background drift speeds, at each of the four drift directions, resulting in 60 trials per block. Participants completed a block of trials for each of the three object speeds, resulting in a total of 180 trials. The design was doubly randomized, as the presentation order of trials within each block was shuffled and the order of blocks was counterbalanced between participants. The experiment took *ca* 35 minutes to complete.

Data processing followed the same procedure as in Experiment 1. Error values equal to, or below, 16 pixels were coded as hit, and those above as miss. Overall, 47.4% of targets were hits and 52.6% were misses. Again, between-participant variation in performance was approximately normally distributed (Shapiro–Wilk: *w* = 0.977, *p* = 0.837), and none were removed as outliers.

GLMMs were fitted, and model output was summarized, using the same procedure as Experiment 1. Maximal random effects were included in the overall main and interaction effects GLMM. However, a singularity warning was given for the post hoc models with maximal random effects owing to close-to-zero variance for the random slopes. Therefore, although a maximal model approach is generally preferable [53], we fitted random intercepts (only) models instead. In fact, the fixed effect estimates from the maximal models were very similar to those from the random-intercept models, but we present the latter because we are more confident about model fit.

## 3. Results and discussion

### 3.1. Experiment 1

#### 3.1.1. Results

The data are shown in figure 3.

**Figure 3.**
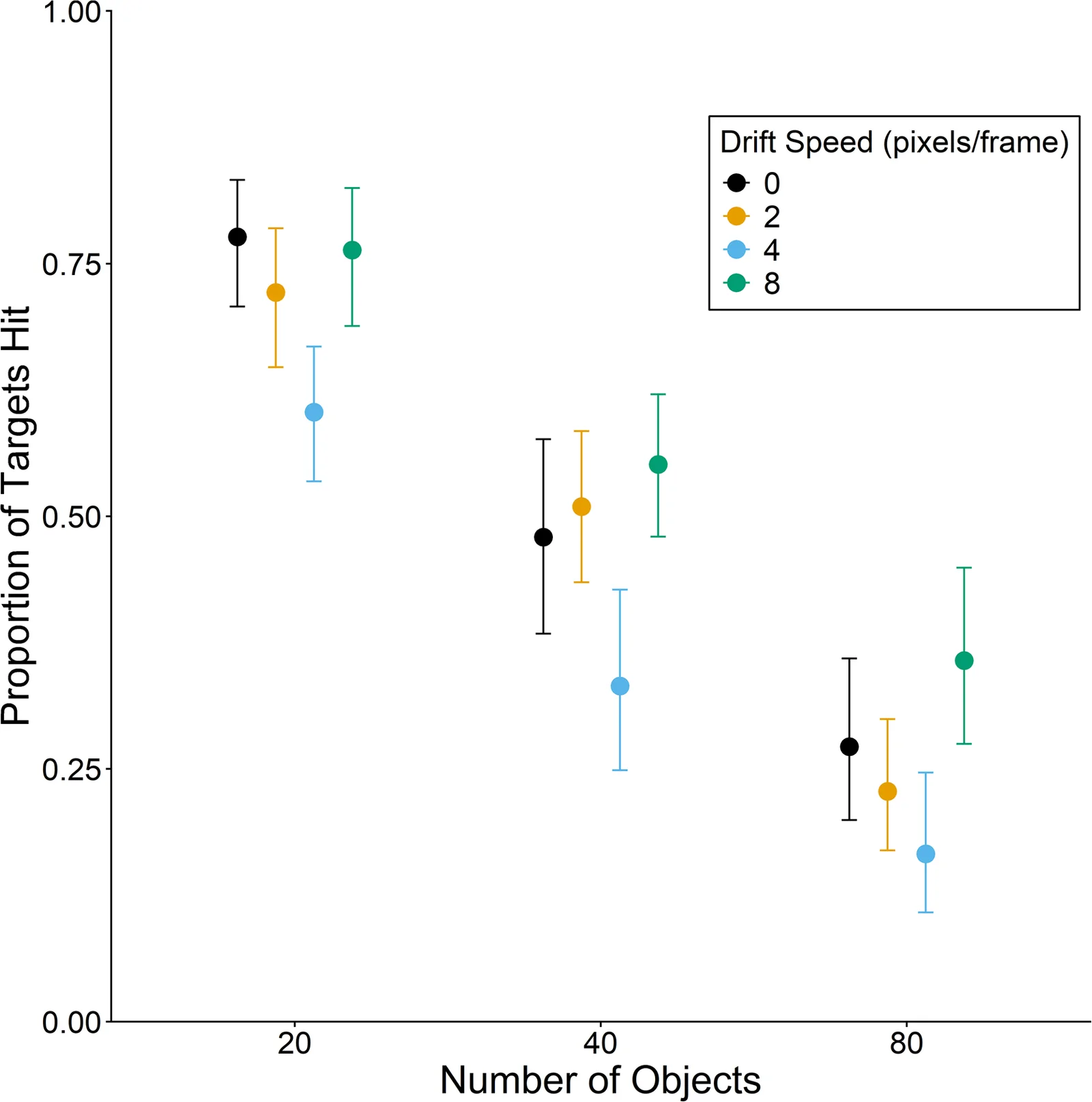
Experiment 1: proportion of hits as a function of group size and background drift speed. Error bars represent 95% confidence intervals for the mean, calculated from the fitted GLMM.

The interaction of group size and background drift speed was non-significant (*χ*^2^ = 4.77, d.f. = 6, *p* = 0.573), but there were significant main effects of group size (*χ*^2^ = 261.09, d.f. = 2, *p* < 0.001) and of background drift speed (*χ*^2^ = 64.07, d.f. = 3, *p* < 0.001). Fitting a polynomial contrast to group size showed that the proportion of hits decreased linearly with each doubling of group size (a log–linear effect of group size; linear coefficient = –1.58, *z* = –9.55, *p* < 0.001; quadratic coefficient = 0.17, *z* = 1.08, *p* = 0.282). Tukey-style pairwise comparisons of background drift speed found that tracking success when backgrounds drifted at 4 pixels per frame was significantly poorer compared with drift speeds of 8 pixels per frame (*z* = –7.86, *p* < 0.001), 2 pixels per frame (*z* = –5.32, *p* < 0.001) and the static control (*z* = –5.68, *p* < 0.001). Tracking was significantly poorer when backgrounds drifted at 2 pixels per frame, compared with 8 pixels per frame (*z* = –3.11, *p* = 0.010). There were no significant differences between drift speeds of 2 and 0 (*z* = –1.09, *p* = 0.697) or 8 and 0 (*z* = 2.14, *p* = 0.140). As the non-significance of the interaction term represents a lack of evidence that drift speed has different effects at each group size rather than evidence that the effect is the same, for completeness [55], Tukey-style pairwise comparisons of tracking success between background drift speeds were conducted at each level of group size (supplementary material).

#### 3.1.2. Discussion

For Experiment 1, we hypothesized that dynamic backgrounds would impede accurate tracking of a target object among distractors, with impairment being greatest when the temporal dynamics of object and background movement matched; that is, when the background moved at the same speed at which objects moved.

Background drift speed influenced tracking, such that tracking success was poorer when the background drifted at 4 pixels per frame compared with other speeds. Since tracking success at background drift speeds of 2 and 8 pixels per frame did not differ from the static background control, the hypothesis that dynamic backgrounds would generally impair tracking compared with static backgrounds was not supported. Instead, tracking was uniquely impaired by drifting backgrounds that moved at the same speed as the objects (4 pixels per frame), which supports the hypothesis that matching the temporal dynamics of objects and their background resulted in the greatest impairment to tracking. A crucial limitation is that, since the object speed did not vary, we cannot be certain that this impairment is due to temporal background matching *per se* or whether this holds only for backgrounds drifting at this speed in particular. This is addressed in the next experiment, where both object and background speed were manipulated in a factorial design.

Tracking accuracy reduced as group sizes increased. This reflects the typical pattern of the confusion effect, the reduced attack-to-kill ratio resulting from difficulty in discriminating an individual target within a group [8–10,15,16]. In addition, the influence of dynamic backgrounds was largely consistent across group sizes (figure 3), even though not all pairwise differences were significant when group sizes were analysed separately (with a concomitant reduction in power; supplementary material).

### 3.2. Experiment 2

#### 3.2.1. Results

The data are shown in figure 4.

**Figure 4.**
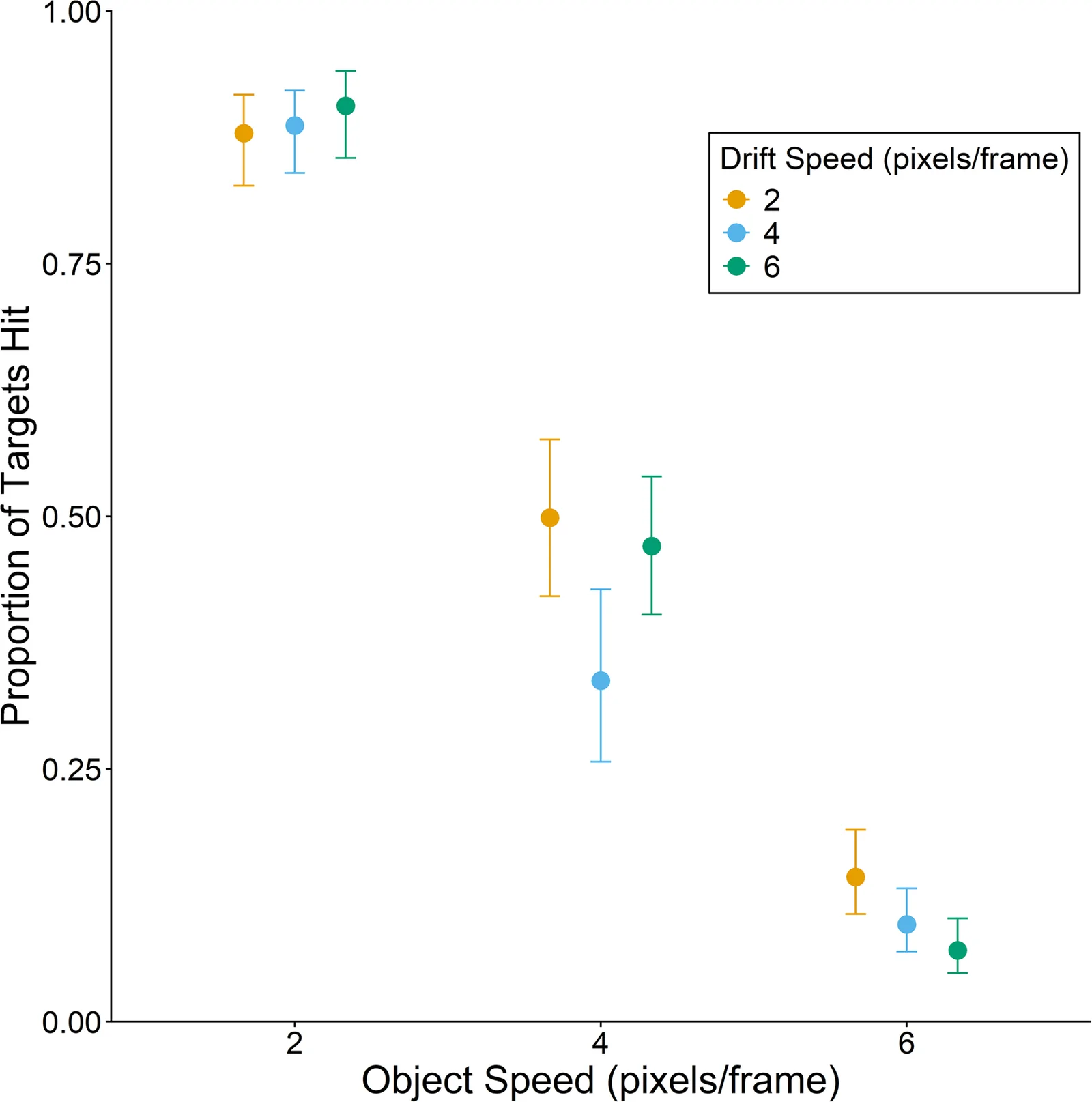
Experiment 2: proportion of hits as a function of object speed and background drift speed. Error bars represent 95% confidence intervals for the mean, calculated from the fitted GLMM.

There was a significant interaction effect between object speed and background drift speed (*χ*^2^ = 17.52, d.f. = 4, *p* = 0.002; main effects of object speed *χ*^2^ = 647.16, d.f. = 2, *p* < 0.001 and drift speed *χ*^2^ = 19.06, d.f. = 2, *p* < 0.001). To explore the cause of the significant drift-by-object speed interaction, Tukey-style pairwise comparisons between background drift speed conditions were conducted within each level of object speed. No significant differences in tracking success were found between drift speed conditions at object speed 2 pixels per frame (*χ*^2^ = 0.83, d.f. = 2, *p* = 0.661). At an object speed of 4 pixels per frame there were significant differences (*χ*^2^ = 23.53, d.f. = 2, *p* < 0.001), with tracking significantly less successful at background drift speed 4 pixels per frame compared with drift speed 2 pixels per frame (*z* = –4.59, *p* < 0.001) and drift speed 6 pixels per frame (*z* = –3.73, *p* < 0.001), with no significant difference between drift speeds 6 and 2 pixels per frame (*z* = –0.87, *p* = 0.657).

In trials where object speed was 6 pixels per frame, background drift speed had a significant effect (*χ*^2^ = 14.82, d.f. = 2, *p* < 0.001), with tracking significantly poorer at a drift speed of 6 pixels per frame compared with drift speed 2 pixels per frame (*z* = –3.70, *p* < 0.001) and 4 pixels per frame compared with drift speed 2 pixels per frame (*z* = –2.42, *p* = 0.041). No significant difference was found between drift speeds of 6 and 4 pixels per frame at this object speed (*z* = –1.36, *p* = 0.363).

#### 3.2.2. Discussion

Experiment 2 aimed to establish whether dynamic backgrounds enhance the confusion effect uniquely when drifting at 4 pixels per frame, or conversely, through a masking effect when the temporal properties of the background and objects’ motion match. The interaction between object speed and background drift speed indicated that the deleterious effects of a drifting background on tracking were not general, but were influenced by the speed at which objects moved. Therefore, the masking hypothesis was best supported by our data. This was evident in the post hoc pairwise comparisons. As in Experiment 1, when objects moved at 4 pixels per frame, tracking was poorer when backgrounds drifted at this same speed, compared with conditions where backgrounds drifted at either 2 or 6 pixels per frame. Tracking was also poorer at an object speed of 6 pixels per frame when backgrounds drifted at the same speed, compared with a speed of 2 pixels per frame. Although there were no differences between background speed conditions when objects moved at 2 pixels per frame, and within the object speed of 6 pixels per frame where tracking success did not differ between background drift speeds of 6 and 4 pixels per frame, this is probably due to floor and ceiling effects at the sampled parameters of object speed. The pattern of data is suggestive of a bandpass masking effect, such that disruption to tracking is greatest when the speed of the dynamic background most closely matches that of the objects and attenuates as speed becomes more mismatched. This is what one would expect on signal-to-noise considerations: the target (signal) must not only be discriminated from similarly moving non-target objects but must also be discriminated from similarly moving background textures.

Object speed was the dominant factor influencing tracking success, consistent with multiple tracking tasks demonstrating reduced tracking capacity and performance with increasing speed [56–58]. Our parameters for object speed were informed by pilot experiments, which observed similar performance at the two extremes but suggested potential for differences between drift speed conditions. We did not sample at finer gradations of object speed because the previous experiment visually suggested a bandpass-like pattern of masking and, as such, sampling speed parameters with sufficient range ensured that a masking effect would not have been obscured. Despite the ambiguity of some effects at the extremes of object speed, the significant interaction between object and background speed does support a non-general masking effect of dynamic backgrounds. Taken alongside the directionality of effects, this suggests that dynamic backgrounds optimally enhance confusion through temporal background matching, when the speed of objects most closely matches that of their background.

### 3.3. General discussion

Investigations into how predation success is affected by dynamic backgrounds are warranted, as motion is pervasive in natural environments and habitats [3]. The two experiments presented here provide evidence that dynamic backgrounds enhance the confusion effect. Because this effect depends on the relationship between object and background motion, our results suggest that enhanced confusion is not simply because the moving background distracts attention. Instead, prey moving in groups may benefit from drifting dynamic backgrounds, through disrupted tracking, but only when the temporal dynamics of the prey group matches those of the background. Experiment 1 demonstrated that tracking of moving objects was impeded when backgrounds drifted at the same speed, with backgrounds drifting faster or slower being no more detrimental than a static control. Experiment 2 found an interaction of object speed and background drift speed, establishing that the disruptive effect of a drifting background was influenced by the speed at which objects moved, with data suggestive of a greater impairment to tracking when objects and backgrounds moved at similar speeds.

The data therefore support the hypothesis that the greatest impairment to tracking would arise from dynamic backgrounds that drift at the same speed as targets and distractors moved. This aligns with the signal-to-noise framework [19], which posits that concealment is contingent on minimizing the salience of a signal (i.e. target) relative to surrounding noise. Dynamic backgrounds increase the amount of noise that must be filtered out in order to accurately track a signal and, logically, the most misleading and disruptive noise would be that which is most similar to the signal [23,24]. Such an interpretation receives further support from observations of behaviours constrained by, or aiming to exploit, dynamic backgrounds in nature. For example, dynamic territorial signals in agamid lizards demonstrate clear contrast from the typical motion noise of their background, which aligns with the aim of making these signalling behaviours conspicuous [59]; and behaviours that apparently aim to conceal against moving backgrounds typically demonstrate temporal properties which closely resemble those of the surrounding motion noise [32,33,60].

Note that our finding that dynamic backgrounds did not generally impede tracking over static backgrounds was contrary to our hypotheses, based on the signal-to-noise framework and previous experimental investigations. For example, Matchette *et al.* [25] found that detection of an individual moving target was both slower and more error-prone when presented on a background with dynamic illumination, containing caustic patterns found in shallow aquatic environments, as opposed to a static scene. Marine and terrestrial patterns of dynamic illumination were similarly observed to disrupt the speed to detect and attack prey in visually guided aquatic and avian predators [29,30]. We therefore expected that, since dynamic backgrounds inherently contain more noise than static backgrounds, they may reduce the signal-to-noise ratio and mitigate the costs of movement to objects over static backgrounds [1–3,5,18,61,62]. This discrepancy between our findings and the literature may arise from fundamental differences in the dependent measures: dynamic backgrounds may generally impede detection and recognition, but not generally impede tracking of a signal once detected, with disruption at this level of processing being contingent on shared properties with the background. However, differences may also relate to the nature of the dynamic backgrounds presented. Dynamic illumination, particularly water caustics, varies randomly and unpredictably in spatial and temporal domains [3,26,28]. Our coherent, directed pattern of movement better represents motion noise arising from some physical properties, such as wind-blown foliage and flowing or tidal water, which may move less randomly. Indeed, more chaotic dynamic noise may be more difficult to filter out and therefore may provide a general impediment to detection or tracking, whereas more coherent motion, as used here, is easier to disregard unless its properties are similar to a target's signal. It is tempting to speculate that this may be done automatically in the visual cortex, where prolonged exposure to a coherently moving stimulus leads to reduced firing rates for directionally selective neurons owing to adaptation [63], an effect related to the so-called motion after-effect or ‘waterfall illusion’ (see [64] for a review).

Along these lines, generalization of our results may be constrained by stimulus specificity. Our drifting background may be applicable to avian predators tracking shoals of fish in flowing freshwater, but it is unclear based on our data whether disrupted tracking of group-living prey could be achieved in other natural patterns of background motion. Experimental investigations using other patterns of motion may be necessary to establish the plausibility of masking for shoals of coral fish by caustics, or terrestrial insects and mammals by dappled light. Natural sources of dynamic noise are also likely to vary in intensity, direction and pattern over short temporal scales, unlike our stimuli, which held these variables controlled and constant. Nevertheless, our results highlight a novel role for dynamic backgrounds in interacting with group movement to impede predation, as well as highlighting the importance of temporal background matching.

It is worth considering the mechanism behind our observed effect. This masking did not arise from perfect and continuous matching of the background's motion; indeed, the objects moved unpredictably in terms of direction, and therefore would have matched the direction of the drifting background only very temporarily. This probably represents a mechanism of temporary masking or concealment, where the target became briefly camouflaged by matching the speed and direction of the background's motion, and subsequently had to be relocated among similar distractors. This is comparable with the findings of [65], where temporary occlusion of a target by similar conspecifics enhanced the confusion effect and hindered target reidentification. The presence of distractors is vital to this mechanism, as an individual target would be easily relocated when synchrony with the background is broken, but the presence of distractors creates difficulty in re-selecting the same target once lost owing to masking. Since the objects had a perfectly background matching texture, it remains to be investigated whether such an effect is contingent on the matching of spatial properties (i.e. visual texture), as well as temporal properties, of the background. It is also likely that this impediment would be mitigated by inter-individual variation in pattern, since phenotypic attributes would become a cue to identity (the so-called oddity effect [66]), facilitating relocation and discrimination from distractors as well as reducing confusion (as was found by [65]).

While our results identify a role for dynamic backgrounds in impeding predation of group-living prey, some important considerations about the generalizability of these experimental data to natural predatory encounters warrant discussion. First, the movement of objects in this experimental task does not reflect the movement of many animals in groups. Group movement in animals can be highly coordinated, as in flocks of starlings (*Sturnus vulgaris)* [67,68], fish schools [69,70] and to some extent in flying insects [71]; however, other animals in groups show little order in heading or coordination and their movement appears erratic, irregular and independent, such as in flying [72–74] and grounded insects [75,76] and some fish aggregations [70]. This latter class of animal movement is much like the random walks used for object movement in the present experiments, and erratic and unpredictable movement is one common example of a ‘protean’ defence used by prey [77]. While some computer-based experiments have examined confusion effects in coordinated and polarized moving groups, e.g. [67,78], most have had prey moving independently and uncoordinated [9,10,65,79,80] and have generated informative insights into factors influencing confusion.

Second, it may be argued that tracking a single designated target for the entire duration of a pursuit is not a realistic constraint for natural predators, who are free to choose any individual in a group. However, the mechanism of confusion, whereby a tracked individual is lost among its moving conspecifics, would affect any given group member. Indeed, fixation on multiple objects within a trial plausibly occurs as one object is lost and another distractor is fixated upon. This is suggested in experimental data where participants were instructed to continuously track a designated target with the cursor [9] and might be more clearly demonstrated using eye-tracking methods or continuous monitoring of the locations of distractors, in addition to the cursor and target, in similar experimental tasks. We would expect that in any pursuit requiring predators to track a given individual in order to achieve capture, though this target may change throughout the encounter, tracking would be deleteriously affected through confusion effects and by dynamic noise in a background, in much the same way as in the present experiment. Of course, some predators may adopt alternative strategies to targeting individuals when hunting group-living prey, such as picking out prey opportunistically, targeting the centre of a group and capturing an individual item once in reach [81,82].

The research presented here also has important implications for how camouflage may operate under dynamic conditions. A long-standing assumption has been that ‘motion breaks camouflage,’ with experimental tasks finding that background matching textures are ineffective at reducing detection rates and latencies for moving targets under static conditions [18,83]. However, Hall *et al.* [18] demonstrated that this assumption is not necessarily true, as motion noise from moving distractors can reduce detection and recognition times for background matching prey, mitigating the costs of movement. Our research builds upon this, suggesting that group-living camouflaged prey can additionally benefit, through reduced predation success, from motion noise in the background with temporal properties that are similar to those of the group's movement.

## Supplementary Material



## Data Availability

Data are available at the University of Bristol data repository, data.bris, at [84]. Supplementary material is available online [85].
