# Supplementary material for: Dynamic backgrounds enhance the confusion effect
Source: R Soc Open Sci. 2026 Oct 28;13(10):261915. doi: 10.1098/rsos.261915 (PMC13714400; doi:10.1098/rsos.261915)
Supplement: Supplementary file 1 [file rsos.261915.zip › rsos.261915/rsos261915_si_001.docx]

**Online Supplementary Materials**

**Dynamic Backgrounds Enhance the Confusion Effect**

Luca C. R. Courtney, Innes C. Cuthill, Ute Leonards, Nicholas E. Scott-Samuel.

*Royal Society Open Science* (doi:10.1098/rsos.261915)

**Table S1.** Post-hoc pairwise comparisons (Tukey) between background drift speeds at each group size in Experiment 1

| Group Size | Background Speed Comparisons | Estimate | z | *p* |
| --- | --- | --- | --- | --- |
| 20 |  |  |  |  |
|  | 2 - 0 = 0 | -0.28 | -1.55 | .405 |
|  | **4 - 0 = 0** | **-0.79** | **-4.47** | **< .001** |
|  | 8 - 0 = 0 | -0.09 | -0.47 | .966 |
|  | **4 - 2 = 0** | **-0.51** | **-2.97** | **.016** |
|  | **4 - 8 = 0** | **-0.70** | **-4.02** | **< .001** |
|  | 8 - 2 = 0 | 0.20 | 1.09 | .697 |
| 40 |  |  |  |  |
|  | 2 - 0 = 0 | 0.10 | 0.65 | .917 |
|  | **4 - 0 = 0** | **-0.58** | **-3.51** | **.003** |
|  | 8 - 0 = 0 | 0.28 | 1.70 | .324 |
|  | **4 - 2 = 0** | **-0.69** | **-4.14** | **< .001** |
|  | **4 - 8 = 0** | **-0.86** | **-5.15** | **< .001** |
|  | 8 - 2 = 0 | 0.17 | 1.05 | .718 |
| 80 |  |  |  |  |
|  | 2 - 0 = 0 | -0.27 | -1.47 | .453 |
|  | **4 - 0 = 0** | **-0.54** | **-2.83** | **.024** |
|  | 8 - 0 = 0 | 0.38 | 2.17 | .130 |
|  | 4 - 2 = 0 | -0.27 | -1.37 | .517 |
|  | **4 - 8 = 0** | **-0.91** | **-4.91** | **< .001** |
|  | **8 - 2 = 0** | **0.65** | **3.62** | **.002** |

Differences in the proportion of errors between treatments are expressed on the logit scale. Significant differences in bold. Singularity warnings were given for models with random effects of (Drift.speed | Participant), due to close-to-zero variance; therefore, models included a random effect of (1 | Participant) instead.
